# Novel Rifampicin and Indocyanine Green Co-Loaded Perfluorocarbon Nanodroplets Provide Effective *In Vivo* Photo–Chemo–Probiotic Antimicrobility against Pathogen of Acne Vulgaris *Cutibacterium acnes*

**DOI:** 10.3390/nano10061095

**Published:** 2020-06-01

**Authors:** Kuang-Hung Hsiao, Chun-Ming Huang, Yu-Hsiang Lee

**Affiliations:** 1 Department of Biomedical Sciences and Engineering, National Central University. No. 300, Jhongda Rd., Taoyuan City 32001, Taiwan; 105827005@cc.ncu.edu.tw; 2 Department of Dermatology, University of California, San Diego, CA 92093, USA; 3 Department of Chemical and Materials Engineering, National Central University, Taoyuan City 32001, Taiwan

**Keywords:** acne vulgaris, perfluorocarbon, nanoemulsion, probiotics, antibiotics, phototherapy, photo–chemo–probiotic therapy, *Cutibacterium acnes*

## Abstract

Acne vulgaris is one of the most prevalent dermatological diseases among adolescents and is often associated with overgrowth of *Cutibacterium acnes* (*C. acnes*) in the pilosebaceous units. In this study, we aimed to develop novel rifampicin (RIF) and indocyanine green (ICG) co-loaded perfluorocarbon nanodroplets named RIPNDs which can simultaneously provide photo-, chemo-, and probiotic-antimicrobility, and explore their efficacy in treatment of *C. acnes* *in vitro* and *in vivo*. The RIPNDs were first characterized as being spherical in shape, with a size of 238.6 ± 7.51 nm and surface charge of −22.3 ± 3.5 mV. Then, the optimal dosages of *Staphylococcus epidermidis*–produced fermentation product medium (FPM) and RIPND were determined as 25% (*v*/*v*) and [RIF]/[ICG] = 3.8/20 μM, respectively, based on the analyses of inhibition zone and cytotoxicity *in vitro*. Through the *in vivo* study using *C. acnes*–inoculated mice, our data showed that the group treated with FPM followed by RIPNDs + near infrared (NIR) irradiation obtained the least granulocytes/macrophage-inflammatory protein 2 expression level in the epidermis, and showed a significantly lower microbial colony population compared to the groups treated with equal amount of RIF, FPM, RIPNDs, and/or combination of the above ± NIR. These results indicated that the RIPND-mediated photo–chemo–probiotic therapeutics was indeed able to rapidly suppress inflammatory response of the skin and provide a robust antibacterial effect against *C. acnes* with limited use of antibiotics. Taken altogether, we anticipate that the RIPND is highly potential for use in the clinical treatment of acne vulgaris.

## 1. Introduction

Acne vulgaris (AV) has long been recognized as one of the most common dermatological diseases that affects >80% of adolescents and young adults worldwide [1]. The pathogenesis of AV is multifactorial. In general, this dermatosis is usually associated with microbial colonization of pilosebaceous units with overgrowth of *Cutibacterium acnes* (*C. acnes*), a Gram-positive anaerobic commensal microorganism formerly named *Propionibacterium acnes*, hyperkeratinization, and obstruction of sebaceous follicles as results of abnormal keratinization of the infundibular epithelium and subsequent perifollicular inflammation [2]. Typically, AV lesions are pleomorphic, ranging from open/closed non-inflammatory comedones to inflammatory papules, pustules, cysts, and/or nodules, and are frequently occurred on seborrheic areas, face, neck, chest, and upper back [3,4].

Nowadays, although a number of chemical administrations, including topical, systemic, and/or a combination of the above, have been applied to AV treatment, the therapeutic effect is still mainly dependent on the antibiotics used. Antibiotics without microbial specificity may unselectively destroy all the bacteria and maintain the homeostasis of the microflora at the lesion site. Some of medicines, such as tetracycline, doxycycline, minocycline, and erythromycin, may be able to further suppress the inflammatory response to relieve pain for patients [5]. However, long-term or overuse of antibiotics may generate microbial drug resistance and/or induce detrimental side effects, such as pigmental change, atrophy, skin irritation, hepatotoxicity, and/or birth defects [6,7,8,9,10], that highly hinder the utilization of antibiotics in the clinic.

To reduce the antibiotic use and/or aim for AV patients who fail traditional chemotherapy, phototherapy is a feasible alternative that has been widely developed in the last decade. Agents such as 5-aminolevulinic acid, methyl aminolevulinic acid, indole 3-acetic acid, and indocyanine green (ICG) have been successfully employed as photosensitizers [11,12,13,14], while red/blue lights, pulsed dye lasers, and intense pulsed light have all been used with good results [15,16,17]. The efficacy of phototherapy on AV treatment is generally thought to be attributed to apoptosis of sebaceous glands, immune modulation, reduction in sebum secretion, and bactericidal effectiveness [18,19]. However, most of the photosensitizers are liable to be disintegrated in physiological/aqueous environment, and such degradation can be greatly accelerated by light irradiation (photo-degradation) and/or heating (thermal-degradation) [20]. For example, ICG appears to be completely bound with serum proteins and rapidly decay in 18 wt.% per minute, following its intravenous administration, resulting in only 2–4 min of half-life in the circulation [21,22]. In addition, a number of issues, including the types of photosensitizers, light sources, operation procedures, and unfavorable side effects after treatment, such as scaling and/or scarring, make the phototherapy complicated and thereby reduce its applicability in the clinic.

In addition to chemo- (antibiotics) and photo-therapeutics, recently skin probiotics has been recognized as a feasible tool for dermatotic treatment [23,24,25]. Similar with the concept of yogurt-mediated gastrointestinal healthcare, the probiotics employed from the skin commensal microflora or external supplements may be able to maintain the ecological balance of the epidermal microbiome and/or suppress the growth of pathogenic microbes, to grant healthy skin [26]. Previously it has been reported that the fermentation products generated from the *C. acnes*, *Staphylococcus epidermidis* (*S. epidermidis*), and *Staphylococcus lugdunensis* were able to inhibit the growth of methicillin-resistant *Staphylococcus aureus* [27], *C. acnes* [28], and *Candida parapsilosis* [29], respectively. A recent study from Wang et al. further showed that the sucrose may be used as an effective agent to selectively induce fermentation of *S. epidermidis* for *C. acnes* inhibition [30]. These efforts suggested that skin probiotics is likely served as a feasible tool for AV treatment. However, over-fermentation of a microorganism may disturb the ecological balance of the commensal microflora at the lesion site (e.g., AV) and cause unforeseen dermatoses, consequently.

To incorporate the merits of each approach without aforementioned drawbacks, we previously developed a new type of nanometer-sized double-layer perfluorocarbon (PFC) nanocarrier encapsulated with rifampicin (RIF) and ICG, named rifampicin (RIF)-ICG-loaded PFC nanodroplets (RIPNDs), and preliminarily demonstrated their multi-bactericidal effect, including chemical (antibiotics), photo (photothermal and photodynamic), and probiotic antimicrobility against *C. acnes in vitro* [31]. Based on the data obtained in the previous study, the RIPNDs were able to (1) significantly improve the thermal stability of the entrapped ICG; (2) generate a tremendously increased amount of singlet oxygen upon NIR exposure compared with that produced by equal amount of ICG; and (3) selectively increase fermentation rate of *S. epidermidis*, therefore showing an enhanced antibacterial efficacy against *C. acnes* compared with RIF alone. To further identify the applicability of the RIPND in the clinic, in this study, the RIPND-mediated photo–chemo–probiotic antibacterial capability was comprehensively investigated through an *in vivo* assay. In addition, more *in vitro* antibacterial effectiveness and cytotoxicity of the RIPND were examined and analyzed in this paper.

## 2. Materials and Methods

### 2.1. Fabrication and Characterization of the RIPNDs

The RIPNDs were manufactured through two emulsification processes, as reported previously [31]. In brief, RIF (0.04 wt.%) and ICG (0.1 wt.%), in 500 μL methanol, were first mixed with perfluorooctyl bromide (PFOB) containing polyethoxylated fluorosurfactant in 2% (w/w) at ambient temperature. The mixture was then homogenized under an ice bath for 5 min to form the primary water-in-PFC emulsions. The produced emulsion medium was immediately added to an aqueous solution containing carboxylic Pluronic F68 copolymer (5% (w/w)), followed by homogenization under an ice bath for 10 min, to carry out the RIPND manufacture. The obtained RIPNDs were washed twice with deionized water and stored in 4 °C, until use.

The surface charge and size distribution of the RIPNDs were measured by using dynamic light scattering (DLS) technique. The morphology of the RIPND was detected by cryo-transmission electron microscopy (cryo-TEM; JEM-1400, Japan Electron Optics Laboratory Co., Ltd., Tokyo, Japan).

### 2.2. Microbial Cultivation

*S. epidermidis* (ATCC^®^ 12228^TM^, ATCC, Manassas, VA, USA) was cultured by using tryptic soy broth (TSB) under aerobic conditions. *C. acnes* (ATCC^®^ 6919^TM^, ATCC) was cultured by using Reinforced Clostridium Medium (RCM) under anaerobic conditions (Gaspak system). Both bacteria were maintained with 200 rpm shaking at 37 °C and quantified by spectrophotometry at *λ*_abs_ = 600 nm. Bacterial clusters were collected by centrifugation at 5000× *g* for 10 min under ambient temperature. Overnight cultures were diluted 100-fold and proceeded cultivation until the spectrophotometric absorbance value (i.e., optical density; OD_600_) of the bacterial sample reached ≥1.0.

### 2.3. Measurement of Antimicrobial Efficacy of the RIPNDs in vitro

The antimicrobility of the RIPND was evaluated, using the inhibition zone approach. In short, *C. acnes* in 50 μL TSB with 1 × 10^6^ colony-forming units (CFUs)/mL was smeared on each RCM agar plate, and a 5 mm filter paper was placed on the center of each plate. Afterward, 10 µL of RIF solution, fermentation product medium (FPM), and RIPNDs ± FPM were separately dropped on the filter papers and subjected to ±NIR exposure, as indicated in Table 1.

In this antimicrobial study, the NIR irradiation was performed, using an 808 nm laser with output intensity of 6 W/cm^2^ for 5 min. The concentrations of RIF in the aqueous solution were corresponding to the dosages provided by the RIPNDs, and all of the concentration settings, including ICG and RIF in the RIPND samples, are shown in Table 1. In this study, the FPM was prepared by co-culturing 1 × 10^9^ CFUs/mL of *S. epidermidis* and the RIPNDs ([ICG]/[RIF] = 1.25/0.24 μM) in rich medium (10 g/L yeast extract, 3 g/L TSB, 2.5 g/L K_2_HPO_4_, 1.5 g/L KH_2_PO_4_, and 0.002% (w/v) phenol red) for 72 h and was collected by centrifugation at 5000× *g* for 30 min. For each group, the clear area around the central filter paper, where there was neither growth nor survival of the bacteria, was quantitatively measured by using the ImageJ software, after incubation under anaerobic conditions at 37 °C for 72 h.

### 2.4. Cell Culture

Human keratinocytes (KERTr cells; ATCC^®^ CRL-2309^™^, ATCC) were cultured, using keratinocyte serum-free medium supplemented with bovine pituitary extract and human recombinant epidermal growth factor, and maintained at 37 °C, with 5% CO_2_ and 100% humidity.

### 2.5. In Vitro Cytotoxicity Assay

To evaluate cytotoxicity of the FPM, 12 mL of culture medium containing 6 × 10^6^ KERTr cells was aliquoted into 6 wells of a 6-well culture plate and incubated at 37 °C for 24 h. Afterward, the cells were separately treated with 0%, 6.25%, 12.5%, 25%, 50%, and 100% (*v*/*v*) of FPM for 24 h and subjected to viability analysis, using hemocytometry with trypan blue exclusion method. The group treated with normal culture medium without FPM was employed as the control.

To examine the cytotoxicity of the RIPNDs and/or RIPND-associated methods, 6.4 mL of culture medium containing 3.2 × 10^6^ KERTr cells was aliquoted into 32 wells of a 96-well culture plate and incubated at 37 °C for 24 h. Afterward, the RIF solutions, RIPNDs, and RIPNDs + FPM were added to 6, 12, and 12 wells, respectively, while the cells in the other two wells were maintained in normal culture medium. In this study, the concentrations of RIF in water were equal to the dosages provided by the RIPNDs and were set as 0.24, 0.47, 0.95, 1.9, 3.8, and 7.6 μM, by which the concentrations of ICG provided by the RIPNDs were 1.25, 2.5, 5, 10, 20, and 40 μM, respectively. The concentration of FPM used in the settings of RIPNDs + FPM was determined based on the results of FPM cytotoxicity described above. After replacing the conditioned media, the cells in the six wells with RIPNDs, six wells with RIPNDs + FPM, and one well without an agent were subjected to NIR irradiation (808 nm; 6 W/cm^2^) for 90 s, followed by incubation at 37 °C for an additional 24 h. The cells in the other 19 wells without NIR treatment were consistently maintained with the conditioned media at 37 °C for 24 h. The viabilities of cells in all groups were analyzed by using MTT assay afterward.

### 2.6. Animal Study

A total of 20 Institute of Cancer Research (ICR) mice (8–12 weeks, Harlan Labs, Placentia, CA, USA) weighing between 25 and 35 g were employed in this animal study. The animal protocols used in this study followed the guidelines approved by the Institutional Animal Care and Use Committee (IACUC) set in the National Central University (Approval number: NCU-106-016; accepted at 19 December 2017). Experimental mice were anesthetized via inhaling isoflurane. Both ears of each ICR mouse were intradermally injected with 10 µL PBS containing 1 × 10^7^ CFUs/mL of *C. acnes*, using an insulin syringe with 29 G × 1/2 inches (BD Biosciences, San Jose, CA, USA). After maintenance in isolated cages for 24 h, the bacteria-inoculated ears were separately treated with NIR, RIF, RIPNDs ± NIR, and FPM ± (RIPNDs ± NIR) under different procedures. In this animal study, the NIR irradiation was performed by using an 808 nm laser with output intensity of 6 W/cm^2^ for 90 s and was operated immediately after the injection of nanodroplets (if there was). The concentration of RIF was equal to the dosage provided by the RIPNDs that was set as [RIF]/[ICG] = 3.8/20 μM. The concentration of FPM was determined based on the results of FPM cytotoxicity described above. FPM ± (RIPNDs ± NIR) denotes that the ears with bacteria were first treated with FPM for 4 h, followed by RIPNDs ± NIR, if there was. All the experimental mice were maintained in the cages for 24 h after treatment, and then they were sacrificed. The appearances of all ears were photographed prior to (1) bacteria injection, (2) drug treatment, and (3) sacrifice. Afterward, the ears were excised, weighed, and homogenized for *C. acnes* quantification and analysis of inflammatory response *in vivo*.

### 2.7. Evaluation of Antimicrobial Effect of the RIPNDs In Vivo

After the mice were sacrificed, the ear homogenate of each group was prepared by using 200 mg of the ear and was collected by centrifugation, at 8000× *g* for 10 min, at 4 °C. The supernatants of homogenates were placed on RCM agar plates and maintained at 37 °C, under anaerobic conditions. In this study, the antimicrobial capability of each treatment was determined based on the value of microbial population index (MPI) gained by counting the colony formation units (CFUs) on the RCM agar plates after 72 h incubation (MPI = Log_10_ ((CFU + 1)/mL)).

### 2.8. Measurement of Inflammatory Response In Vivo

In this study, the degree of inflammatory response of each group was quantitatively determined based on the level of the macrophage inflammatory protein-2 (MIP-2) expressed in the ear homogenate. The expression level of MIP-2 was detected by using the Mouse CXCL2/MIP-2 Immunoassay kit (R&D System, Inc., Minneapolis, MN, USA), in association with spectrophotometry at λ = 450 nm, according to the manufacturer’s instruction. The concentration of MIP-2 of each group was calculated through interpolation analysis of the absorbance value (OD_450_), using the standard curve of OD_450_ vs. MIP-2 concentration (pg/mL) set prior to the experiment.

### 2.9. Histological Study

The ears around the bacterial inoculation sites were harvested by sharp dissection after the mice were sacrificed. The tissues were dehydrated in graded ethanol and fixed by formalin, followed by xylene clearance and paraffin embedding for a routine histological process. A 5 μm thick section obtained from each paraffin block was stained by hematoxylin and eosin (H&E) and subjected to microscopic image analyses, using the Motic DSA Viewer software (Version 1.0. Motic Asia, Kowloon, Hong Kong).

### 2.10. Statistical Analysis

All data were acquired from multiple independent experiments (*n* ≥ 3) and are presented as the mean ± standard deviation (SD). Statistical analyses were conducted using MedCalc software, (Version 19.0.7. MedCalc Software Ltd., Ostend, Belgium) through which comparisons for one condition between two groups were performed by Student’s *t*-test, with a significance level of *p* < 0.05 throughout the study.

## 3. Results and Discussion

### 3.1. Morphological and Physicochemical Analyses of the RIPNDs

**Figure 1:**
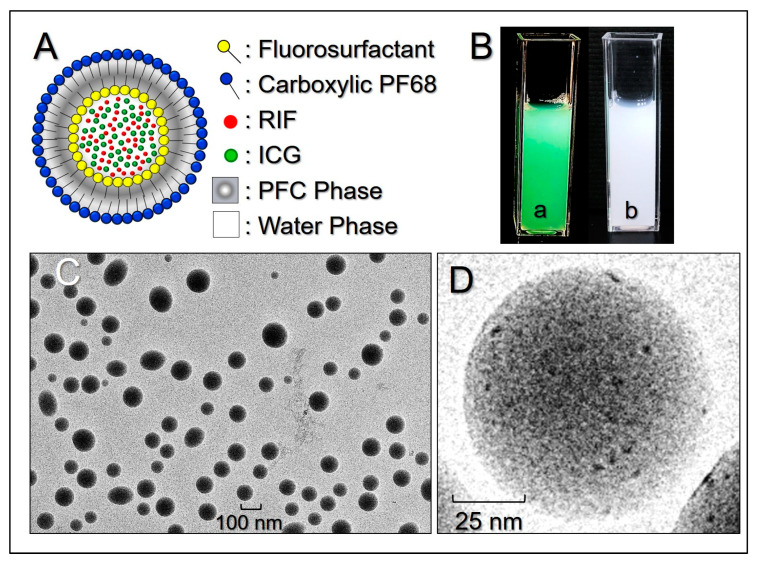
Morphological and physicochemical analyses of the RIPND. (**A**) Schematic diagram of the structure of the RIPND. (**B**) Photographs of the RIPND (a) and blank PFC nanoemulsion (b) samples. (**C**) Cryo-TEM image of the RIPNDs at 1500× magnification. (**D**) Cryo-TEM image of a single RIPND at 80,000× magnification.

Figure 1A shows the theoretical double-layer structure of the RIPND and indicates the location of each component, including PFOB, ICG, and RIF inside the nanodroplet. Such double-layer configuration was formed based on the continuous/discontinuous phase allocation of water and PFC during the twice emulsification processes reported previously [31]. The green-to-orange emulsified appearance of the RIPNDs (Figure 1B(a) illustrates the existence of ICG and RIF in the nanodroplets compared to the milk-like blank PFC emulsions (Figure 1B(b). Based on the cryo-TEM detection, the RIPNDs were all in particulate shape (Figure 1C) with rough and porous surface (Figure 1D), showing that the produced nanocarriers were all maintained in intact configuration, without collapse after being through the tough fabrication procedures, such as high-speed centrifugation and/or agitation. The mean size of the RIPND was 238.6 ± 7.51 nm with a polydispersity index of 0.09–0.14, and the surface charge was about –22.3 ± 3.5 mV, according to the DLS measurement. In addition, the encapsulation and loading rates of ICG in the RIPND were 96.4 ± 2.32% and 0.58 ± 0.19 wt.%, respectively, while those of RIF were 68.7 ± 7.68% and 0.14 ± 0.03 wt.%, respectively, according to the UV-vis spectrophotometric analysis.

### 3.2. Antibacterial Effect of the RIPNDs In Vitro

**Figure 2:**
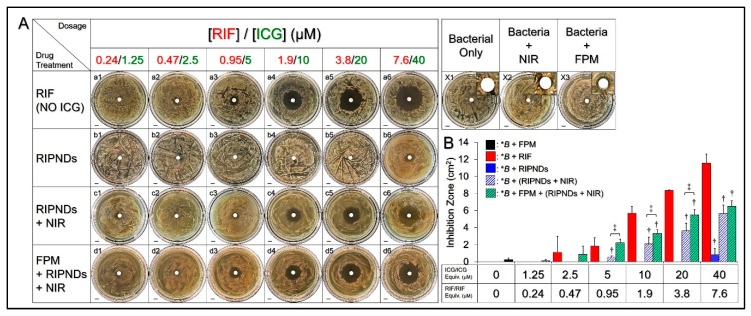
Antimicrobial effects of various treatments against *C. acnes in vitro*. (**A**) Photographic images of inhibition zones of *C. acnes* generated by using NIR (X2), FPM (X3), RIF (row a), RIPNDs (row b), RIPNDs + NIR (row c), and FPM + RIPNDs + NIR (row d). X1 denotes the group without any treatment (blank). The inset images in X1–X3 are the magnified photographs showing the growth conditions of bacteria around the central filter papers. NIR exposure was performed by using an 808 nm laser with output intensity of 6 W/cm^2^ for 5 min. The concentrations of RIF used in the RIF-solution-treated group (row b) were equal to the dosages provided by the RIPNDs, and those were set as [RIF]/[ICG] = 0.24/1.25, 0.47/2.5, 0.95/5, 1.9/10, 3.8/20, and 7.6/40 μM, as indicated in the figure. All groups were photographed, using a digital camera, after incubation under anaerobic conditions at 37 °C for 72 h. Scale bar = 5 mm. (**B**) Quantitative analyses of the inhibition area shown in (A). Values are mean ± SD (*n* = 3). ^*^B denotes the bacteria used in the inhibition zone assay (i.e., *C. acnes*). ^†^ *p* < 0.05 compared to the group with equal dose of RIF ^‡^*p* < 0.05.

Figure 2A shows the formation of inhibition zones in *C. acnes* plates 72 h after various treatments. The concentrations of RIF solutions (Figure 2A, a1–a6) used in the antimicrobial experiments were corresponding to the dosages provided by the RIPNDs. Through the quantitative analyses of the inhibition area (Figure 2B), our data showed that the group with FPM (Figure 2A/X3), but not the one with NIR irradiation (Figure 2A/X2), exhibited a visible 0.23 cm^2^ inhibition zone (Figure 2B), indicating that the FPM produced from the *S. epidermidis* was indeed able to provide an inhibitory effect against *C. acnes* growth, while merely NIR laser irradiation was nontoxic to the bacteria. On the other hand, a dose-dependent antibacterial effect could be obtained in each drug-treated group (Figure 2A; rows a–d), and the results showed that (1) the RIF can generate a noticeable inhibition zone, since the concentration was ≥0.95 μM (Figure 2A, a3–a6); and (2) the treatment of FPM followed by RIPNDs + NIR irradiation (Figure 2A, row d) enabled a higher inhibitory effect to *C. acnes* growth throughout the dose range compared with use of RIPNDs (Figure 2A, row b) or RIPNDs + NIR irradiation (Figure 2A, row c). In this study, the FPM was used to generate the hypothetic probiotic effect on *C. acnes* growth, whereas the setting of FPM + RIPNDs + NIR was designed to mimic the treatment of RIPND-mediated photo–chemo–probiotic therapy operated on a human skin.

Through the comparisons for whole groups, the setting with ≥1.9 μM RIF exhibited the largest inhibition zone among all groups with equal dosage. We speculated that it was because the naked antibiotics were able to provide a faster and immediate antibacterial efficacy under such *in vitro* setup, while the RIPNDs can provide a significant antibacterial effect, only in association with NIR irradiation (Figure 2A, b4 vs. c4). In addition, we found that the ICG-led phototherapy can dramatically enhance the antibacterial effect of the RIPNDs, and the resulted inhibition area was even bigger than that caused by using a four-fold higher amount of RIPND without NIR (Figure 2A, c4 vs. b6). Besides, the FPM can further promote the antibacterial effect of the RIPNDs + NIR, whereby the inhibition area was significantly enhanced by 4.2- (*p* < 0.05), 1.6- (*p* < 0.05), 1.5- (*p* < 0.05), and 1.2-fold, when the dose of RIF in the RIPND were set as 0.95, 1.9, 3.8, and 7.6 μM, respectively (Figure 2B). These results indicated that the RIPNDs combined with FPM and NIR were certainly able to inhibit the growth of *C. acnes*.

### 3.3. Cytotoxicity of the FPM and RIPNDs In Vitro

**Figure 3:**
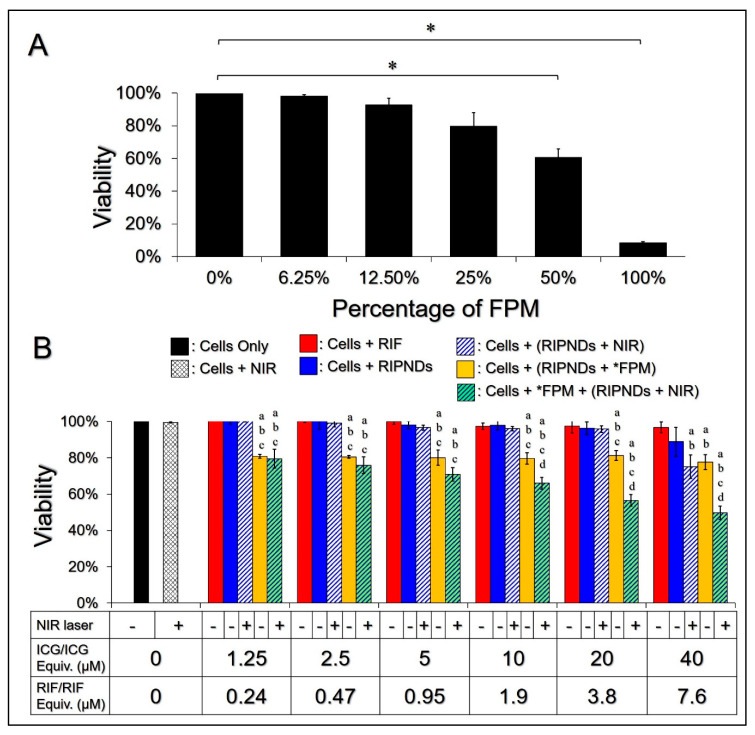
Cytotoxicity of the FPM and the RIPNDs to human skin cells *in vitro*. (**A**) Viabilities of the KERTr cells after treatment with 0% (cell culture medium only), 6.25%, 12.5%, 25%, 50%, and 100% (*v*/*v*) of FPM for 24 h. Values are mean ± SD. *—*p* < 0.05. (**B**) Viabilities of the KERTr cells 24 h after various treatments, as indicated in the *x*-axis. Values are mean ± SD (*n* = 3). NIR exposure was performed by using an 808 nm laser with output intensity of 6 W/cm^2^ for 90 s. *—FPM was employed in concentration of 25% (*v*/*v*). a*—p* < 0.05 compared to the group with equal dose of RIF. b*—p* < 0.05 compared to the RIPND-treated group with equal dosage of RIPND. c*—p* < 0.05 compared to the (RIPNDs + NIR)-treated group with equal dosage of RIPND. d*—p* < 0.05 compared to the (RIPNDs + FPM)-treated group with equal dosage of RIPND.

The applicability of the RIPND was evaluated by not only the antibacterial efficacy, but also its toxicity to the mammalian cells. In this study, the cytotoxicity of the FPM with various concentrations to KERTr cells were first examined, using the MTT assay, and the results were shown in Figure 3A. It was obtained that the cytotoxicity of the FPM was in a dose-dependent manner, by which the cell viability could be maintained in ≥80% when the concentration of FPM was ≤25% (*v*/*v*), whereas it may dramatically decrease to < 60% as the FPM was elevated to ≥50% (*v*/*v*). These results indicated that the FPM in ≤25% (*v*/*v*) was less toxic to cells, and this dosage (i.e., 25% (*v*/*v*)) was used in the following experiments.

Figure 3B shows the viabilities of KERTr cells 24 h after treatment with various conditions. According on the MTT analysis, ≥90% of the cells with NIR, RIF, or RIPNDs through the dose ranges survived, indicating that the moderate photo and/or chemical impacts generated from the aforementioned treatments were nontoxic. The viabilities of the cells treated with FPM (25% (*v*/*v*)) + RIPNDs under various dosages of RIPND were all similar to that with 25% FPM alone (~80%, Figure 3A), showing that the cytotoxicity of FPM + RIPNDs was mainly attributed to FPM. In addition, a remarkable dose-dependent cytotoxicity can be obtained in the groups with FPM + RIPNDs + NIR or RIPNDs + NIR, in which the cells in the former treatment using RIPNDs with ≥10/1.9 μM [ICG]/[RIF] exhibited a significantly higher mortality rate compared with all the other groups, as plotted in Figure 3B (*p* < 0.05 for all the comparisons).

Considering the unfavorable conditions to drug carriers, such as low particle diffusion, enzymatic attack, and multiple drug clean-up mechanisms, including reticuloendothelial system and transcapillary filtration when they were used *in vivo*, the RIPNDs were predicted to be off-target and/or quickly removed from the body; therefore, the amount of RIPND used for AV treatment was suggested to be maximized according to the results of *in vitro* analysis. To obtain a roust antibacterial efficacy with ≥60% cell viability, the dosage of RIPND with 20 μM ICG and 3.8 μM RIF was used in the subsequent animal study.

### 3.4. Anti-Inflammatory Response of the RIPNDs In Vivo

**Figure 4:**
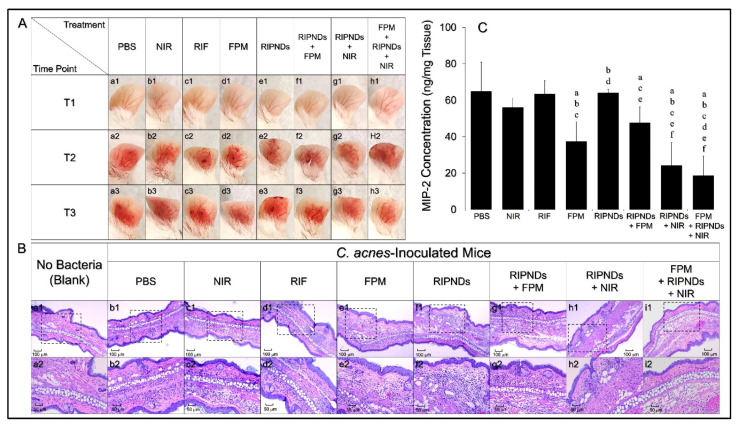
Assessment of inflammatory responses in *C. acnes*–inoculated mice after various treatments. (**A**) Appearances of the mouse ears photographed at three different time points; T1: before injection of *C. acnes* (the original condition); T2: 24 h after *C. acnes* inoculation without treatment; T3: 24 h after treatment (before the mice were sacrificed). Columns a–h denote the different treatments as indicated in the figure. The concentration of RIF used in the RIF-solution-treated group was equal to the dosage provided by the RIPNDs, and that was set as [RIF]/[ICG] = 3.8/20 μM. The FPM was used by concentration of 25% (*v*/*v*). NIR exposure was performed by using an 808 nm laser with output intensity of 6 W/cm^2^ for 90 s. (**B**) Photomicrographic images of H&E-stained ear tissues after various treatments. The photographs of a2–i2 are the magnified images of the area marked by the dash blocks shown in a1–i1. Columns a–i denote the different treatments, as indicated in the figure. (**C**) Quantitative analysis of the MIP-2 expression levels in the mice ears 24 h after treatments. Values are mean ± SD (*n* = 5). Letters a, b, c, d, e, and f denote *p* < 0.05 compared to the MIP-2 concentration values gained from the groups with PBS, NIR, RIF, FPM, RIPNDs, and RIPNDs + FPM, respectively.

The antimicrobial effect of the RIPND with optimized dosages of ICG, RIF, and FPM against *C. acnes* in mice were investigated after *in vitro* antibacterial and cytotoxicity examinations. Figure 4A shows the appearances of the mouse ears before (Figure 4A, a1–h1) and 24 h after (Figure 4A, a2–h2) *C. acnes* inoculation, as well as 24 h after treatment (Figure 4A, a3–h3) for each group. It can be seen that intradermal injection of *C. acnes* may induce a serious inflammation in the whole ear tissue, and the treatment of FPM + RIPNDs + NIR can dramatically ameliorate the inflammatory response after 24 h, based on significantly reduced redness and swelling of the ear (Figure 4A, h2 vs. h3).

Similar results could be found in the histological analysis. Figure 4B shows the H&E staining images of all ear tissues harvested after the animals were sacrificed. It can be observed that the group with FPM + RIPNDs + NIR (Figure 4B, column i) exhibited the least amount of mononuclear cells in the epidermal layer, compared with the other seven bacteria-treated groups (Figure 4B, column b–h), and that was even similar with the condition shown in bacterium-free group (Figure 4B, column a). These outcomes indicated that the method of FPM, followed by RIPNDs + NIR, was the most effective approach to suppress *in vivo* inflammatory response among the eight different treatments, and that was consistent to the results gained from mouse ear observation (Figure 4A). In addition, no obvious tissue damage was observed for all groups, indicating that the treatments of ICG-mediated phototherapy, chemicals, probiotics, and/or combination of above employed in this study were less toxic to the ear skin.

In this study, the significant inflammatory response caused by the inoculation of *C. acnes* was characterized as the lesion of epithelioid macrophages, and that was usually bounded by lymphocyte cuff [32]. In the event of severe AV, *C. acnes* may move into the dermal layer once the follicular wall was ruptured and induce serious inflammatory response subsequently [33]. Therefore, intradermal injection of *C. acnes* into mouse ears may represent an animal model for the granulomatous type of inflammatory AV. To quantitatively evaluate the inflammatory degree of each group after treatment, the expression level of MIP-2, the murine counterpart of human interleukin 8, in each ear tissue, was further measured immediately after the mice were sacrificed. The average weight of mice ears was about 45.08 ± 4.14 mg. As shown in Figure 4C, it can be seen that the group with FPM showed a significantly decreased MIP-2 level compared with that gained from the PBS-treated group (*p* < 0.05), suggesting that the FPM produced from the *S. epidermidis* was able to reduce the inflammatory response caused by *C. acnes* inoculation. Given that butyric acid, one of the key elements in FPM [30], is the class I histone deacetylase (HDAC) inhibitor [34] and may also be able to perform as a ligand of free fatty acid receptor 1 (Ffar1; also known as G protein-coupled receptor 40, GPR40) [35], we reasoned that the FPM-mediated anti-inflammation was achieved through inhibition of HDAC and/or activation of Ffar1, because either mechanism was able to suppress the chemokine induction in the keratinocytes and therefore attenuated cutaneous inflammatory response, as reported previously [36,37,38]. Furthermore, our data showed that the group with FPM + RIPNDs + NIR exhibited the lowest MIP-2 expression among the eight settings, by which the level (18.7 ± 10.64 ng/mg) was approximately 70.5% (*p* < 0.05), 50% (*p* < 0.05), 70.8% (*p* < 0.05), 60.8% (*p* < 0.05), and 23.1% less than the values gained from the groups with RIF, FPM, RIPNDs, RIPNDs + FPM, and RIPNDs + NIR, respectively. These results clearly showed that the treatment of RIPNDs combined with FPM and NIR irradiation (i.e., FPM + RIPNDs + NIR) was able to provide a rapid anti-inflammatory effect to *C. acnes* infection that is highly desirable for AV treatment in the clinic.

### 3.5. Antimicrobial Effect of the RIPNDs In Vivo

**Figure 5:**
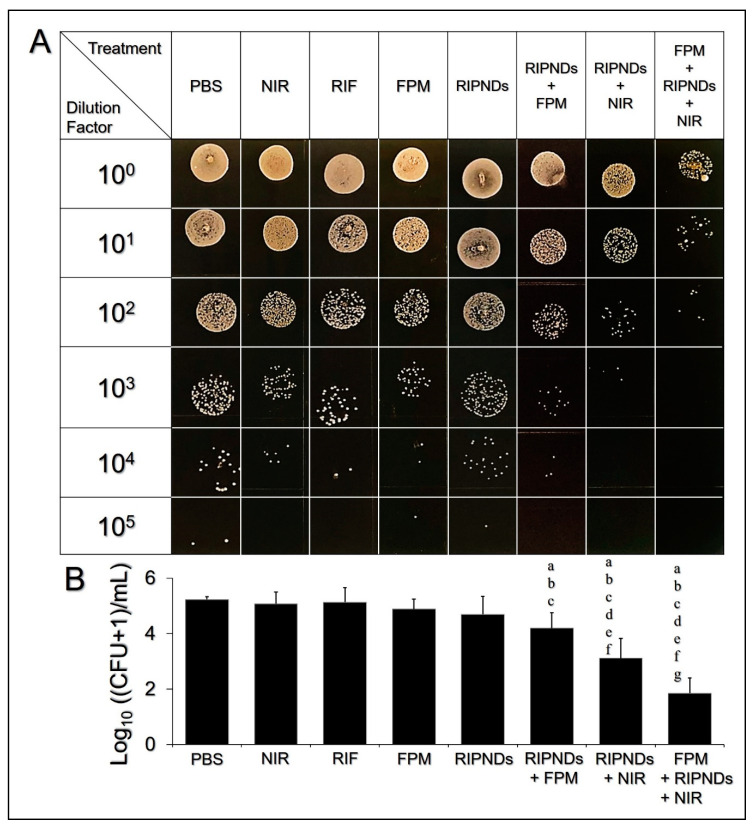
Antimicrobial effects of various treatments against *C. acnes in vivo*. (**A**) Photographic images of the *C. acnes* colonies after various treatments. The six rows represent the colony conditions made by using 10^0^–10^5^-fold diluted bacteria as the seed. The concentration of RIF used in the RIF-solution-treated group was equal to the dosage provided by the RIPNDs and that was set as [RIF]/[ICG] = 3.8/20 μM. The FPM was used by concentration of 25% (*v*/*v*). NIR exposure was performed by using an 808 nm laser with output intensity of 6 W/cm^2^ for 90 s. All colony images were photographed by using a digital camera after cultivation on the RCM agar plates under anaerobic conditions for 72 h. (**B**) Quantitative analyses of the MPI of all groups shown in (A). Values are mean ± SD (*n* = 5). Letters a, b, c, d, e, f, and g denote *p* < 0.05 compared to the MPI values gained from the groups with PBS, NIR, RIF, FPM, RIPNDs, RIPNDs + FPM, and RIPNDs + NIR, respectively.

The *in vivo* antimicrobial effect of the RIPND was examined through the measurement of the MPI of the *C. acnes* isolated from the ear tissues after the mice were sacrificed. Figure 5A exhibits the results of bacterial colony formation of all groups after incubation on the RCM agar plates for 72 h. It can be seen that the one with FPM + RIPNDs + NIR exhibited a remarkably lower colony population number compared with the other seven groups. Based on the MPI analyses (Figure 5B), the microbial populations of the groups with treatments of NIR (MPI = 5.08 ± 0.43), RIF (MPI = 5.14 ± 0.53), FPM (MPI = 4.89 ± 0.36), and RIPNDs (MPI = 4.71 ± 0.65) were all similar to the one using PBS (MPI = 5.24 ± 0.11; *p* = NS for all), indicating that the aforementioned treatments were all insufficient to provide an effective antimicrobility *in vivo*, whereas the operations of RIPNDs + FPM, RIPNDs + NIR, and FPM + RIPNDs + NIR enabled a significant antibacterial effect compared to the one using PBS or RIF alone (*p* < 0.05 for all comparisons). Based on the MPI analysis, the group with FPM + RIPNDs + NIR exhibited the highest bactericidal efficacy among the eight settings.

In the mice study, the RIF (3.8 μM) and FPM (25% (*v*/*v*)) exhibited similar *in vivo* bactericidal effects based on the MPI analysis (Figure 5B, *p* = NS). In contrast to their markedly distinct antimicrobilities in the inhibition zone tests (Figure 2), we speculated that 3.8 μM of RIF can merely provide bacteriostatic rather than bactericidal effect to *C. acnes*, according to the minimal bactericidal concentration (MBC) of RIF (~5 μM) reported previously [39]. Furthermore, both RIF and FPM may be quickly cleaned up by reticuloendothelial system and/or transcapillary filtration and thus resulted in a deficient *in vivo* antibacterial effect for the two settings, as illustrated in Figure 5. In addition, both *in vitro* (Figure 2) and *in vivo* (Figure 5) data showed that the RIPNDs were less toxic to *C. acnes*, but the RIPNDs combined with NIR may serve a robust bactericidal agent, showing that the phototherapy indeed played a pivotal role in the RIPND-mediated anti–*C. acnes* treatment. Moreover, the antibacterial effectiveness achieved by RIPNDs + NIR can be further enhanced by pretreatment of *C. acnes* in FPM (i.e., FPM + RIPNDs + NIR). In this animal study, the FPM was used to mimic the hypothetic probiotic effect on human body, since the *S. epidermidis* are intrinsically distributed on the human skin surface and can be rapidly fermented due to presence of RIPNDs as reported previously [31]. Based on the MPI analysis (Figure 5B), the subcutaneous *C. acnes* with 4 h pretreatment of FPM, followed by RIPNDs + NIR, suffered a 40% (*p* < 0.05) higher death rate compared with those treated by RIPNDs + NIR, indicating that the FPM/probiotics played an adjuvant but significant role in the RIPND-mediated anti–*C. acnes* treatment. However, the RIF-led chemotherapy from the RIPNDs is indispensable, because it may take over the therapeutic tasks from the FPM and ICG after NIR exposure and provide a continuous antimicrobial effect thereafter. Taken all together, these results clearly showed that the RIPND-mediated photo–chemo–probiotic therapy can provide an enhanced antimicrobial efficacy under limited use of RIF, and thus may be able to generate less antibiotics-induced toxicity or adverse effects found in the clinic.

## 4. Conclusions

In this study, the RIPND-mediated antibacterial effectiveness against *C. acnes* was comprehensively investigated through both *in vitro* and *in vivo* assays. With merits of rapid suppression of inflammation, robust bactericidal capacity, and less requirement of antibiotics for use *in vivo*, the RIPND, which enables photo-, chemo/antibiotic-, and probiotic therapeutics, is considered to be a novel antibacterial agent against *C. acnes*, and such a composite antimicrobial approach may provide a new insight for AV treatment in the clinic.

## Figures and Tables

**Table 1:** The dose settings of the RIF and RIPNDs used for the inhibition zone experiment.

Dosage	NIR	FPM	[RIF]/[ICG] (μM)					
Treatment			#1	#2	#3	#4	#5	#6
^*^ *B*	−	−	-					
	+	−						
	−	+						
^*^B + RIF	−	−	0.24/-	0.47/-	0.95/-	1.9/-	3.8/-	7.6/-
^*^B + RIPNDs	−	−	0.24/1.25	0.47/2.5	0.95/5	1.9/10	3.8/20	7.6/40
	+	−						
	+	+						

^*^B denotes the bacteria (i.e., *C. acnes)* used in the experiments.

## Abbreviations

AV	Acne vulgaris
CFU	Colony forming units
Ffar1	Free fatty acid receptor 1
FPM	Fermentation product medium
GPR40	G protein-coupled receptor 40
HDAC	Histone deacetylase
H&E	Hematoxylin and eosin
ICG	Indocyanine green
MBC	Minimal bactericidal concentration
MIP-2	Macrophage inflammatory protein-2
MPI	Microbial population index
NIR	Near infrared
OD	Optical density
PFC	Perfluorocarbon
PFOB	Perfluorooctyl bromide
RCM	Reinforced clostridium medium
RIF	Rifampicin
RIPND	Rifampicin-indocyanine green-loaded perfluorocarbon nanodroplets
